# Alternative and Experimental Therapies of *Mycobacterium abscessus* Infections

**DOI:** 10.3390/ijms21186793

**Published:** 2020-09-16

**Authors:** Michal Meir, Daniel Barkan

**Affiliations:** 1 Pediatric Infectious Diseases Unit, The Ruth Rappaport Children’s Hospital, Rambam Health-Care Campus, Haifa 31096, Israel; MI_MEIR@rambam.health.gov.il; 2 Koret School of Veterinary Medicine, The Robert H. Smith Faculty of Agriculture, Food and Environment, The Hebrew University of Jerusalem, Rehovot 76100, Israel

**Keywords:** *Mycobacterium abscessus*, drug treatment, experimental therapy, review

## Abstract

*Mycobacterium abscessus* is a non-tuberculous mycobacterium notoriously known for causing severe, chronic infections. Treatment of these infections is challenging due to either intrinsic or acquired resistance of *M. abscessus* to multiple antibiotics. Despite prolonged poly-antimicrobial therapy, treatment of *M. abscessus* infections often fails, leading to progressive morbidity and eventual mortality. Great research efforts are invested in finding new therapeutic options for *M. abscessus.* Clofazimine and rifabutin are known anti-mycobacterial antibiotics, repurposed for use against *M. abscessus*. Novel antimicrobials active against *M. abscessus* include delamanid, pretomanid and PIPD1 and the recently approved beta-lactamase inhibitors avibactam, relebactam and vaborbactam. Previously unused antimicrobial combinations, e.g. vancomycin–clarithromycin and dual beta-lactam therapy, have been shown to have synergistic effect against *M. abscessus* in experimental models, suggesting their possible use in multiple-drug regimens. Finally, engineered phage therapy has been reported to be clinically successful in a severe case of disseminated *M. abscessus* infection. While many of these experimental therapeutics have shown activity against *M. abscessus* in vitro, as well as in intracellular and/or animal models, most have little if any evidence of effect in human infections. Clinical studies of *M. abscesssus* treatments are needed to reliably determine the value of their incorporation in therapeutic regimens.

## 1. Introduction

Non-tuberculous mycobacteria (NTM) are ubiquitous environmental organisms being increasingly recognized as human pathogens, with rising incidence of infections [1]. Of NTMs isolated, *Mycobacterium abscessus* is associated with the most severe infections including progressive pulmonary disease (especially in patients with cystic fibrosis), skin and soft tissue, central nervous system and disseminated, often fatal disease. 

Treatment of *M. abscessus* infections is remarkably challenging. *M. abscessus* is intrinsically resistant to multiple antimicrobials including the anti-tuberculous drugs, and macrolide resistance in the subspecies *abscessus* and *bolletii* [1]. In addition, the chronic nature of *M. abscessus* infections, as well as prolonged sub-lethal concentrations of antimicrobials, drives induced (mutation based) antibiotic resistance, further limiting antibiotic choices and requiring multi-antimicrobial therapy. Even when the effective concentration of antimicrobials is well above the MIC, *M. abscessus* killing is limited due to antibiotic tolerance, especially related to biofilm formation [2,3], therefore adding difficulty in the successful treatment of these patients. 

There is growing evidence that specific *M. abscessus* genotypes are associated with distinct antimicrobial resistance patterns—*erm (41)* and *rrl* gene mutations with macrolide resistance, *rrs* gene mutations with amikacin resistance and *gyrA* and *gyrB* with quinolone resistance [4,5]. Genetic susceptibility patterns are now increasingly recognized as predictors of antibiotic effect versus failure, therefore guiding choice of antimicrobial drugs, especially macrolides and aminoglycosides [6]. 

Genetic studies of *M. abscessus* isolates have enabled recognizing three distinct *M. abscessus* subspecies—*M. abscessus subsp. massiliense*, *M. abscessus subspp. bolletii* and *M. abscessus supsp. Abscessus—*that differ with respect to specific *erm(41)* features and intrinsic clarithromycin susceptibility patterns [7,8,9]. Most isolates of *M. abscessus subsp. abscessus* and *M. abscessus subsp. bolletii* have a full-length and functional *erm(41)* gene, conferring intrinsic resistance to macrolides. In contrast, most *M. abscessus subsp. massiliense* have a truncated, non-functional *erm(41)* gene and are therefore inherently susceptible to macrolides, yet may develop acquired (inducible) macrolide resistance [8,9]. Considering the susceptibility patterns of the *M. abscessus* subspecies, current clinical recommendations include subspecies identification in pulmonary infections with *M. abscessus* in order to guide choice of antimicrobial therapy [1]. 

Current treatment recommendations of *M. abscessus* pulmonary infections include combination therapy of two or more intravenous drugs (i.e., amikacin, tigecycline, imipenem and cefoxitin) with one or two oral antimicrobials including macrolides, linezolid, clofazimine and, occasionally, a quinolone. Choice of antimicrobials is generally guided by in vitro susceptibility testing, and, when available, *erm(41)* and *rrl* genotyping for macrolide susceptibility [1,6,10]. In constitutively resistant strains, macrolides are not recommended as a mainstay of treatment. When treating *M. abscessus* isolates with inducible macrolide-resistance, practice guidelines may differ—while some recommend their use [6], others suggest using other antimicrobials as guided by susceptibility testing [1]. These differences in approach stem from the paucity of clinical data comparing the clinical effect of different treatment combinations. Prolonged multi-antimicrobial therapy is often limited by drug-induced toxicity (such as bonemarrow suppression by linezolid, liver toxicity by tigecycline, development of hypersensitivity to β-lactams, etc.), yet, even under strict regimens, treatment failure rates remain high with recurrent or chronic infections and grave clinical outcome. In accordance with the search for new therapeutics, there is a surge in the number of experimental antibiotics with potential activity against *M. abscessus* in various mechanisms. This review summarizes evidence of novel and experimental therapeutic options for treatment of *M. abscessus* infections. These include novel antibiotics, new—and sometimes counter-intuitive—antibiotic combinations, re-purposing of known antibiotics and phage therapy.

### 1.1. Clofazimine

Clofazimine is a fat-soluble riminophenazine dye that was developed in the 1950s, mainly for treating leprosy, and found to have antibiotic activity against *M. abscessus* isolates. Several studies have shown in vitro synergy between clofazimine and other antibiotics, such as clarithromycin, amikacin, tigecycline and bedaquiline (BDQ) [11,12,13,14], while other studies report possible promotion of resistance [11]. Clinical data on the efficacy of clofazimine are available yet limited. In a recent retrospective report of 42 patients with *M. abscessus* pulmonary infection, sputum culture conversion was achieved in 43% of cases following combination treatment that initially included clofazimine, and in 15% of non-responsive cases (cases in which previous antibiotic treatments failed) [15]. Another cohort study demonstrated favorable outcomes using clofazimine to treat *M. abscessus* pulmonary infection in immune-compromised hosts, yet included only a small numbers of patients [16]. Studies using clofazimine to treat *M. abscessus* infections are summarized in Table 1. Current clinical treatment guidelines recommend clofazimine as a preferred drug for treatment of *M. abscessus* pulmonary infection, although its practical use may be limited by limited availability in many countries, including in the United States [1,10]. 

### 1.2. Bedaquiline 

Bedaquiline (BDQ; code names TMC2017 and R207910) is a diarylquinoline antibiotic. In *Mycobacterium tuberculosis*, it was shown to act through inhibition of the ATP Synthase [48], which is considered true in other mycobacteria as well. It is now recommended by the WHO for use as a part of an antibiotic-combination regimen for multidrug resistant tuberculosis [49]. Reports on in vitro efficacy of BDQ on clinical isolates of *M. abscessus* [50,51] showed most isolates to have an MIC to BDQ ranging 0.016–1 µg/mL, yet a substantial proportion (15% of isolates) had MICs of 16 µg/mL and more. Preclinical in vivo models of *M. abscessus* infection showed variable results. In nude mice infected with the reference strain ATCC 19977, BDQ had no effect on survival or on mycobacteria load [25], while, in GKO^−/−^ mice, SCID mice and zebrafish models, BDQ had a protective clinical effect [13,24].

There are scant data on the clinical effect of BDQ in humans infected with *M. abscessus*, although current reports are somewhat encouraging, namely showing a tolerable safety profile in a multi-drug regimen [20]. However, given a report of an antagonistic effect of bedaquiline with β-lactams [21], caution is warranted when considering this treatment combination. 

### 1.3. Rifabutin

The rifamycin rifabutin has been recently shown to have in vitro activity against reference strains as well as clinical isolates of *M. abscessus.* This antimicrobial activity was approximately 10-fold greater than the activity of rifampin and rifapentine, and it was evident in clarithromycin-resistant strains [27]. Rifabutin was found to be synergistic to clarithromycin against isolates with an intact *erm(41)* gene, and it has been shown to suppress inducible clarithromycin resistance [29], suggesting this drug combination may be of great clinical benefit. 

Recent studies have demonstrated the antimicrobial effect of rifabutin against *M. abscessus* in preclinical models. In a macrophage *M. abscessus* infection model, rifabutin was shown to reduce intracellular bacterial burden and chord formation [30], while, in a zebrafish infection model, rifabutin treatments improved larval survival [30]. In a NOD SCID mouse model, rifabutin was shown to be as effective as clarithromycin in decreasing bacterial *M. abscessus* burden in the spleen and lungs [31]. No clinical trials of refabutin for treating *M. abscesssus* infection are yet available. 

### 1.4. Novel β-Lactamase Inhibitors

Resistance of *M. abscessus* to β-lactams is mediated by multiple mechanisms, including a chromosomally-encoded Ambler-Class A β-lactamase (Bla_Mab_), which is not inhibited by clavulonic acid, tazobactam or sulbactam [52]. In fact, these β-lactamase inhibitors are themselves substrates of the potent Bla_Mab_. The possible activity of newly developed β-lactamase inhibitors has been examined in several recently published studies. Avibactam is a non β-lactam β-lactamase inhibitor approved in combination with ceftazidime for treating Gram-negative bacterial infections. Unlike clavulonic acid and tazobactam, avibactam appears to inhibit Bla_Mab_ [53]. Combining avibactam with amoxicillin or piperacillin was shown to be effective against *M. abscessus* reference strains and clinical isolates, as well as in vivo in zebrafish and *Galleria mellonella* models, respectively [17,53]. In both models, the addition of avibactam rendered amoxicillin and piperacillin to be as effective as meropenem. Surprisingly, avibactam was also found to improve the in vitro and in vivo effect of imipenem, a carbapenem supposedly unaffected by Bla_Mab_ [54], suggesting avibactam may have some subtle intrinsic activity (see the discussion of dual β-lactam treatment below). 

Relebactam and Vaborbactam are other non-β-lactam, β-lactamase inhibitors recently approved for use in the combinations imipenem–relebactam and meropenem–vaborbactam. Relebactam was shown to inhibit Bla_Mab_ and rendered clinical *M. abscessus* isolates susceptible to amoxicillin [55]. Another study examined the effect of relebactam and vaborbactam on the MIC of several carbapenems, (including imipenem and meropenem) and cephalosporins (including cefepime, ceftaroline and cefuroxime) in *M. abscessus* clinical isolates. With the exception of cefoxitin, the MICs of all antibiotics tested decreased in the presence of either relebactam or vaborbactam, suggesting a possible benefit to their use as a part of a β-lactam based combination [18]. Unfortunately, no clinical studies are yet available to assess the efficacy of avibactam, relebactam or vaborbactam in treatment combinations for *M. abscessus* infections. In addition, all the novel β-lactamase inhibitors are currently clinically available only as parts of a fixed ratio drug combination with β-lactams. As both drug-ratio and choice of β-lactams may not be optimal for treating *M. abscessus*, clinical use of the novel β-lactamase inhibitors for this purpose may be complicated.

### 1.5. Dual β-Lactams

The pharmacological principle of using two β-lactams is based on the selective or relatively selective inhibition of non-redundant target enzymes in mycobacterial physiology. β-lactams act by inhibiting transpeptidases essential for the biosynthesis of the bacterial cell-wall. It is now evident that, while most bacteria utilize mostly D,D-transpeptidases (also known as penicillin-binding proteins), mycobacteria rely considerably on L,D-transpeptidases, and that there are several different transpeptidases in each bacteria, each one inhibited to a different extent by various β-lactams. As each β-lactam exerts different inhibitory activity on different L,D-transpeptidases and D,D-transpeptidases, the combination of two β-lactams may have a synergistic effect [56]. Avibactam may also directly inhibit L,D-transpeptidases [57], which may explain why its addition to imipenem is more effective then imipenem alone. Several dual-β-lactam combinations have indeed shown synergy against clinical isolates in vitro, i.e., imipenem with cefoxitin or cefdinir, as well as with avibactam [22]. Similar synergy was shown in a murine chronic pulmonary infection model [23]. Unfortunately, no clinical trials of dual-β lactam therapy in *M. abscessus* treatment are available. However, these studies suggest that using two β-lactam agents in a therapeutic multi-drug regimen may be of benefit rather than redundant, even though historically this regimen may appear counter-intuitive. 

### 1.6. Vancomycin/Clarithromycin 

Vancomycin is a tricyclic glycopeptide antibiotic commonly used against Gram-positive bacteria yet considered ineffective against mycobacteria. Surprisingly, vancomycin was shown to exhibit synergism with clarithromycin against *M. abscesseus* strains that were initially susceptible to clarithromycin [26]. In strains in which clarithromycin resistance was experimentally induced, the addition of vancomycin lowered the MICs to clarithromycin. Conversely, following prolonged exposure to clarithromycin, clinically relevant clarithromycin MICs were not reached, even with the addition of vancomycin, suggesting cautious interpretation when applying this in vitro study to clinical practice [26]. Considering the side effects of prolonged vancomycin treatment, the need for parenteral administration, and the concern for emerging vancomycin-resistant bacteria, clinical use of this combination is deferred pending further evidence. 

### 1.7. Novel Antimicrobials

Omadacycline, a novel aminomethylcycline antimicrobial agent and a member of the tetracycline class of drugs, was recently approved by the U.S. Food and Drug Administration (FDA) for treatment of skin and soft tissue infections and pneumonia [32]. Eravacycline (a fluorocycline) is a new tetracycline analog approved for the parenteral treatment of complicated intraabdominal infections [34]. In vitro studies have shown both omadacycline and eravacycline to have similar antimicrobial activity to tigecycline against both reference and clinical *M. abscessus* strains [32,33,35]. Specifically as omadacycline is available as an oral formulation [58,59], it may have a role in treating chronic *M. abscessus* infections in outpatient settings. Treatment of chronic *M. abscessus* pulmonary infection has been recently reported in one patient, with good tolerability and some clinical benefit [36]. No other clinical trials describing the use of omadacycline or eravcycline in *M. abscessus* infections are available.

Tedizolid is a next-generation oxazolidinone antibiotic approved in 2014 by the FDA for treatment of skin and soft tissue infections [37]. Several in vitro studies have demonstrated antimicrobial activity of tedizolid against *M. abscessus*, alone and combined with other antimicrobials such as clarithromycin and amikacin [37,38,60]. Using a macrophage model, tedizolid was shown to have intracellular antimicrobial activity when used alone, and more so when combined with imipenem with or without avibactam [19]. Compared to the oxazolidinone linezolid, tedizolid is reported to have a more favorable tolerability profile in a 14-day treatment regimen [61]. Clinical reports of treating *M. abscessus* infection with tedizolid are extremely limited. Of note, one report described successful tedizolid treatment of an *M. abscessus* infection in an immunocompromised host [39]. 

Two newly developed anti-tuberculous drugs, delamanid and pretomanid (PA-824), have been recently evaluated for their effect against *M. abcessus*. One study examining MICs of clinical *M. abscessus* isolates found most strains to be resistant to delamanid [62]. Another in vitro study showed that, while pretomanid inhibited the growth of *M. tuberculosis*, it had poor activity against *M. abscessus* [63]. More in vitro and in vivo studies are needed to determine whether delamanid and pretomanid may be of use for treating *M. abscessus* infections. 

Several experimental drugs currently in development have been evaluated for their effect on *M. abscessus*: Delpazolid (LCB01-0371) is a novel oxazolidinone currently in Phase II clinical study for treatment of tuberculosis (available online: https://clinicaltrials.gov/ct2/show/NCT02836483). In a study by Kim et al., delpazolid was shown to have an antimicrobial effect against *M. abscessus* in vitro and in an intracellular macrophage model [40]. In a murine model of infection using high antimicrobial dosage, delpazolid was more effective than linezolid in the lungs but less effective in the spleen and liver [40]. VXc-486 is a novel aminobenzimidazole which targets gyrase B, being evaluated as an anti-mycobacterial drug [64]. VXc-486 was found to potently inhibit growth of *M. abscessus* in vitro (MIC_50_ of 1.0 μg/mL and MIC_90_ of 4.0 μg/mL). In vivo data on VXc-486’s effect on *M. abscessus* infection is lacking, but its potency was demonstrated in a tuberculosis murine model [64]. 

PIPD1, [GSK1985270A; 4-(4-chloro-3-(trifluoromethyl)phenyl)-1-(2-methylbenzyl)piperidin-4-ol], is a new piperidinol-based molecule that acts against mycobacteria by disrupting mycolic acid translocation from the cytoplasm to the periplasmic side of the plasma membrane, disabling the formation of the outer part or mycobacterial cell wall [41]. PIPD1 was shown to exhibit potent activity against clinical *M. abscessus* strains in vitro (MIC of 0.125 μg/mL, bactericidal in time-killing assays), in infected macrophages and in a zebrafish infection model [41]. Indole-carboxamides also act by disrupting mycolic acid transport and production, therefore inhibiting the synthesis of the mycobacterial cell wall [42]. Indole-carboxamides were shown to have a strong antibacterial activity against a wide panel of *M. abscessus* isolates in vitro and in infected macrophages [42], were shown to have synergistic effect with imipenem and cefoxitin [43] and were found active in a murine *M. abscessus* infection model [44]. No clinical trials are available for these experimental drugs. 

MmpL3 inhibitors are a large group of experimental drugs, aimed at several mycobacteria, including *M. abscessus*. The role of MmpL3 in the physiology of the bacteria has only recently been elucidated, and it appears to function as the flippase in mycobacteria—essential in the construction of the cell wall. This group of novel drugs is discussed extensively in another review in this Special Issue, and we therefore do not elaborate on it.

### 1.8. Inhaled Nitric Oxide

Nitric oxide (NO) is produced endogenously and plays an integral role in the host-defense response against bacterial infection [65]. NO has inherent antimicrobial properties against various microorganisms, including bacterial, fungi and parasites [65], and it has been shown to be effective against bacterial biofilms [66]. In addition, NO was found to have a key immune-modulatory role in the defense against mycobacterial infection [67,68]. Several trials have demonstrated the potential use of inhaled NO to treat pulmonary infections, especially in chronic lung disease or infections with *Pseudomonas aeruginosa* in which biofilm formation is considered part of the physiological process [69,70]. Unfortunately, NO had limited therapeutic effect in *Mycobacterium tuberculosis* infection [71]. A recent clinical report of two patients with cystic fibrosis and *Mycobacterium abscessus* infection treated with inhaled NO had promising results, showing a significant reduction in estimated sputum bacterial load [45]. A following open-label study described nine patients with cystic fibrosis and *Mycobacterium abscessus* infection who were treated for 14 days with inhaled NO. This pilot study demonstrated inhaled NO to be a safe, noting no adverse effects. Disappointingly, despite some reduction in sputum bacterial-loads following treatment, findings were not statistically significant, and no significant improvement in lung function was noted in treated patients [46]. Although inhaled NO may have a role in treating chronic lung infections in general, more clinical studies are needed to evaluate its specific effect against *M. abscessus*.

### 1.9. Phage Therapy

The notion of using phages against bacterial infection has been revisited in the past years as part of the ongoing search for solutions for multi-drug resistant organisms [72]. Therapeutic bacteriophages are appealing considering they are pathogen specific and are safe to human tissues [72]. A report of successful bacteriophage treatment of a multi-drug resistant Gram-negative infection has encouraged further studies and clinical trials in this field [73]. In 2019, Dedrick et al [47] reported a case of a 15-year-old lung-transplant patient who suffered from disseminated *M. abscessus* infection, mostly focused to her lungs and skin, whose infection progressed despite all available treatments. The patient received a prolonged treatment of a combination cocktail of three engineered mycobacriophages, and subsequently cleared the infection. No adverse effects were noted for this treatment [47]. It should be noted that most, if not all, characterized mycobacteriophages do not have substantial activity against *M. abscessus*, and the three phages used in this patient were “created“ in laboratory conditions either by “phage training” (repeated passage of the phage in *M. abscessus* until natural selection leads to a phage adapted to the new host) or by directed mutagenesis of the phage, leading to similar results. Hopeful as it may be, phage therapy for *M. abscessus* infection is at this point far from being a practical solution. Phage therapy requires personalized engineering of phages along with a large collection of bacteriophages only available in specific research laboratories, making commercial production impractical. Specifically, the three-phage cocktail used in this study was found to be ineffective against other isolates of *M. abscessus*, thus making it a highly patient-tailored approach and impractical in most clinical settings. In addition, emerging phage-resistance may be a future issue, especially in prolonged treatments [72]. 

## 2. Discussion

Treating *M. abscessus* infections is extremely challenging due to complex antimicrobial resistance profiles; multiple bacterial mechanisms leading to tolerance, thus promoting acquired resistance over time; and limited clinical predictability of in vitro results, all leading to frequent treatment failures despite prolonged multi-drug regimens. As part of a global search for therapies for multi-drug resistant bacteria, new antimicrobials and antimicrobial combinations are evaluated in general and specifically for *M. abscessus*. Unfortunately, most studies examining these antimicrobial agents are either performed in vitro or in cell or animal models (see Table 1). Clinical experience with novel drugs or the optimal drug combinations are scant, leaving physicians to tailor antimicrobial treatment for *M. abscessus* mostly based on MIC values of the bacteria. While there is a dire need for clinical trials comparing treatments, these may be difficult to standardize given the complexity of antimicrobial regimens. In the current medical trend toward personalized medicine, pathogen specific treatment—such as engineered phage therapy or tailored drug-combinations according to combined antimicrobial efficacy against a clinical isolate—may be the key to eradication and clinical success. Whether using a tailored or universal guideline approach, clinical studies are needed to aid treatment decisions for these devastating and chronic infections. 

## Figures and Tables

**Table 1:** Experimental therapies of *Mycobacterium abscessus* infections—evidence summary.

Therapy (Route of Administration)	In Vitro Evidence	In Vivo Models	Published Clinical Experience
Clofazimine (PO)	Synergy with CLR, AMK, TIG and BDQ [11,12]	Treatment of *M. abscessus* in GKO^−/−^ and SCID mice with a combination of CFZ and BDQ was effective [13].	Retrospective study of 42 patients [15], Cohort study in immune-compromised patients. [16]
Bla_Mab_ inhibitors Avibactam Relebactam Vaborbactam (IV)	Active against reference and clinical isolates when combined with β-lactams [17,18,19].	Avibactam combinations effective in macrophage, Zebrafish and *Galleria mellonella* models [20,21].	N/A
Dual β-lactams	Synergy of two β-lactams shown in reference and clinical strains [22]	Synergy in a murine model of chronic pulmonary infection [23]	N/A
Bedaquiline (PO)	Activity in vitro in clinical strains [24] Possible antagonism with β-lactams [21]	Effect of CFZ/BDQ in GKO^−/−^ and SCID mice [13]. No effect in nude mice [25], Protective effect in zebrafish [24].	Report of 10 patients, favorable tolerability [20]
VAN/CLR combination	Synergy of VAN and CLR in reference and clinical strains, questionable effect in strains with acquired CLR resistance [26].	N/A	N/A
Rifabutin (PO)	Activity against clinical and reference strains, including CLR resistant strains [27,28] Synergy with CLR, suppresses CLR induced resistance [29].	Effect in a macrophage model [30], improved survival in a zebrafish model [30], effect similar to CLR in a NOD SCID mouse model [31].	N/A
Omadacycline (PO/IV), Eravacycline (PO/IV)	omadacycline [32,33] and eravacycline [34,35] have activity against reference and clinical strains	N/A	Report of one patient—noted clinical improvement [36]
Tedizolid (PO/IV)	Tedizolid has in vitro alone and combined with CLR and AMK [37,38]	Intracellular effect in a macrophage model [19].	Report of one immune-compromised patient [39]
Delpazolid (PO/IV)	Active against reference strain and 8 clinical strains. Noted spontaneous resistance to delpazolid [40]	Intracellular effect in a macrophage model [40] Comparable effect of delpazolid to linezolid in a murine model [40].	
VXc-486	Active against multiple strains of *M. abscessus*	N/A	N/A
PIPD1	Activity against clinical strains [41]	Intracellular effect in macrophages, effective in a zebrafish model [41].	N/A
Indole-carboxamides	Activity against clinical strains [42] Synergy with imipenem and cefoxitin [43]	Intracellular effect in macrophages [42], effect in a murine model [44].	N/A
Inhaled NO	N/A	N/A	Report of 2 patients with cystic fibrosis showed reduction in sputum bacterial-loads [45]. Report of 9 patients had limited effect [46].
Phage therapy	Profound use in mycobacterial laboratory research	N/A	Treatment of disseminated infection in one patient [47]

Clofazimine, CFZ; Clarithromycin, CLR; Amikacin, AMK; Tigecycline, TIG; Bedaquiline, BDQ; Vancomycin, VAN; Rifabutin, RFB; Nitric Oxide, NO; Not applicable, N/A.

## Abbreviations

NTM	Non-tuberculous mycobacteria
BDQ	Bedaquiline
BLA_MAB_	β lactamase inhibitor of *Mycobacterium abscessus*, Ambler-Class A
CFZ	Clofazimine
CLR	Clarithromycin
AMK	Amikacin
TIG	Tigecycline
VAN	Vancomycin
RFB	Rifabutin
NO	Nitric oxide
FDA	U.S. Food and Drug Administration
